# Molecular design and application of luminescent materials composed of group 13 elements with an aggregation-induced emission property

**DOI:** 10.1093/nsr/nwab049

**Published:** 2021-03-30

**Authors:** Shunichiro Ito, Masayuki Gon, Kazuo Tanaka, Yoshiki Chujo

**Affiliations:** Department of Polymer Chemistry, Graduate School of Engineering, Kyoto University, Kyoto 615-8510, Japan; Department of Polymer Chemistry, Graduate School of Engineering, Kyoto University, Kyoto 615-8510, Japan; Department of Polymer Chemistry, Graduate School of Engineering, Kyoto University, Kyoto 615-8510, Japan; Department of Polymer Chemistry, Graduate School of Engineering, Kyoto University, Kyoto 615-8510, Japan

**Keywords:** aggregation-induced emission, crystallization-induced emission, chromism, conjugated polymer, group 13

## Abstract

Complexation of π-conjugated ligands by metal or semimetal ions leads to the enhancement of the planarity and rigidity of π-conjugated systems. Boron, especially, has played a central role in the design of luminescent main-group complexes. However, these complexes still suffer the disadvantage of aggregation-caused quenching as well as typical organic fluorophores. It has recently been reported that some types of boron complexes exhibit the aggregation-induced emission (AIE) property. Moreover, AIE behavior from complexes and organometallic compounds composed of the other group 13 elements, such as aluminum and gallium, has emerged in this decade. These observations greatly encourage us to develop advanced functional materials based on the group 13 elements. Indeed, recent research has demonstrated that these classes of materials are potentially versatile scaffolds for constructing chromic luminophores, efficiently emissive π-conjugated polymers and so on. This review mainly describes AIE-active group 13 complexes with four-coordinate structures and their application as photo-functional materials. Proposed mechanisms of the origins of AIE behavior are briefly discussed.

## Teaser

Group 13 elements and various ligands have been getting together and paving a new way to develop advanced solid-state luminescent materials with aggregation-induced emission.

## INTRODUCTION

**Figure 1.**
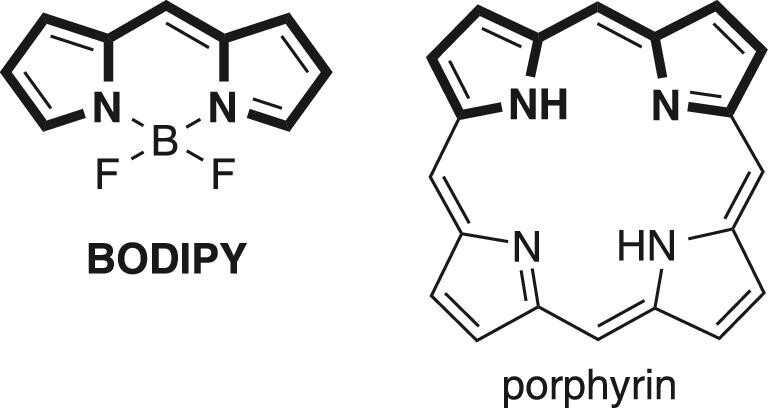
The chemical structure of BODIPY and porphyrin. The common structure is emphasized.

To obtain optimal luminescent properties from organic compounds, chemists have developed various types of π-conjugated compounds over the last 150 years, such as polyaromatic hydrocarbons [1–3], cyanines [4–6], coumarins [7], etc. These luminescent molecules with superior properties possess rigid, planar and often large π-conjugated electronic systems. Such structures generally lead to strong coloration and efficient photoluminescence. In this context, the complexation of π-conjugated ligands with metal or semimetal elements has recently been utilized to construct highly emissive molecules. π-Conjugated ligands locked by coordination bonds result in efficient luminescence in many cases because the complexation enhances molecular rigidity, planarity and effective π-conjugation length. For instance, 4^′^-difluoro-4-bora-3a,4a-diaza-*s*-indacene (BODIPY) and its derivatives, whose dipyrrin ligands can be regarded as a substructure of porphyrins (Fig. 1), exhibit strong absorption and extraordinarily high fluorescence quantum yields in the solution state. BODIPY dyes also exhibit many superb properties, e.g. redox activity, electron transfer and near-infrared emission [8,9].

However, these classes of luminescent dyes often face a severe problem in that many of these complexes lose their emission in the solid or concentrated states due to self-absorption and intermolecular interactions, which give rise to the formation of non-luminescent excitonic states. Such a phenomenon is known as concentration quenching or aggregation-caused quenching (ACQ). The planar structures of the complexes derived from the complexation cause this drawback, although such structural features play a pivotal role in achieving the splendid emission properties. When luminescent materials are applied to anti-counterfeit inks, film-type sensors, light-emitting diodes or other situations requiring a high concentration of dyes, they need to emit efficiently in the concentrated or solid states. Therefore, the trade-off between the complexation and the solid-state luminescence should be circumvented.

Since the aggregation-induced emission (AIE) effect was conceptualized for siloles and tetraphenylethenes, many types of AIE-active compounds have been reported [10–12]. Most of the AIE-active molecules contain bulky and movable substituents, such as phenyl groups, attached to their chromophoric π-systems. These substituents consume excited energy through radiationless processes in the solution states, for example, molecular motions or vibrations. These non-luminescent processes can be suppressed by structural restriction in the concentrated states. The substituents also restrict undesired intermolecular interactions leading to the formation of dark states. Thus, the aggregated AIE-active dyes emit more efficiently than the diluted ones. This significant concept of AIE has shed new light on the molecular design strategy of luminescent boron complexes as well as purely organic luminophores. Indeed, growing numbers of boron-containing molecules and polymers with AIE behavior have been reported over the last decade [12–15]. In addition, some complexes show a crystallization-induced emission (CIE) property. The crystals of CIE-active materials exhibit higher photoluminescence quantum yields than both their amorphous solids and their dilute solutions. Utilizing these classes of compounds, many researchers have developed stimuli-responsive materials, luminescent sensors, emissive films based on AIE-active polymers and so on. Moreover, some examples of the AIE- and CIE-active compounds containing aluminum or gallium have also been explored gradually.

**Figure 2.**
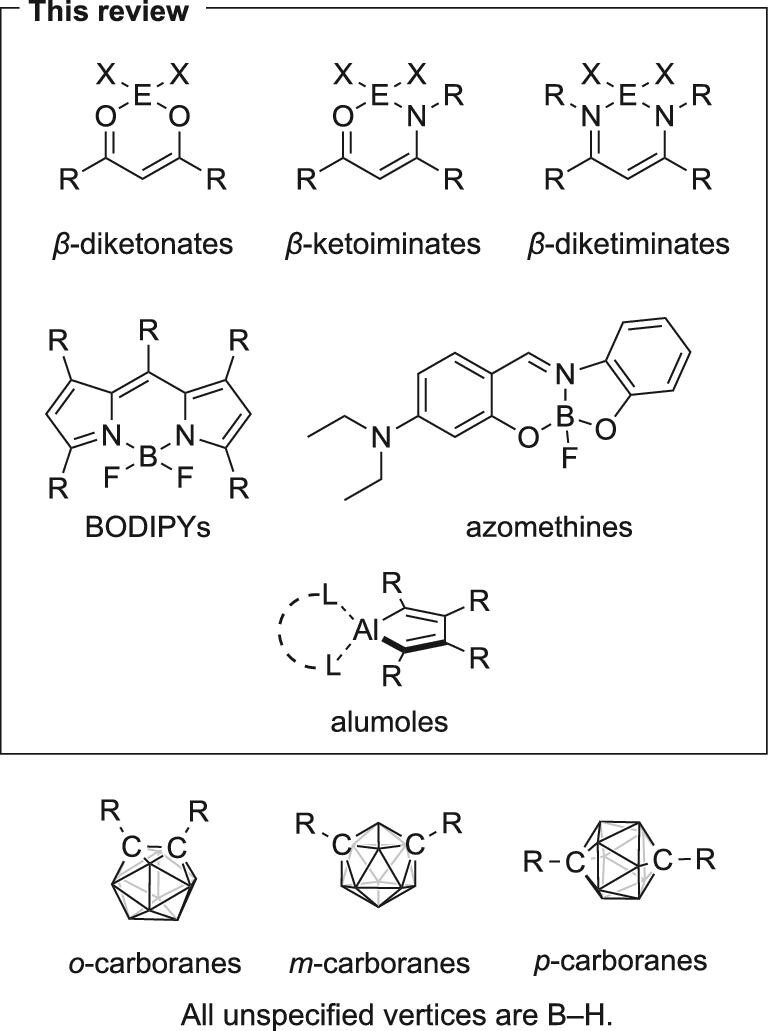
Chemical structures of various scaffolds of AIE-active materials composed of group 13 elements (E = B, Al or Ga; R = aryl, alkyl etc.; X = halogen, aryl etc.).

Heteroatoms, including not only nonmetals but also semimetals, typical, and transition metals, often serve as a key to functionalizing both small molecules and polymers thanks to the promising electronic nature of these atoms. In this context, advanced functional materials can be achieved in a bottom-up manner by elaborately designing a minimal functional unit using heteroatoms. We have regarded such a minimal functional unit as an ‘element block’ [13–15]. Based on this idea, it has been discovered that various types of boron complexes and cluster compounds work as versatile element blocks for realizing AIE behavior. This review mainly focuses on four-coordinate complexes of group 13 elements with AIE and/or CIE properties (Fig. 2). In addition to the coordination compounds, carboranes, which are cluster compounds composed of 10 boron and two carbon atoms, are also known to be a scaffold for AIE-gens. Carborane-based systems have been covered in recent comprehensive reviews [12,16] and will not be mentioned.

## BORON-CONTAINING COMPOUNDS

Four-coordinate boron complexes are usually more stable with regard to air and water than three-coordinate ones because the 2p orbitals of four-coordinated boron are no longer vacant [17]. Consequently, four-coordinate boron complexes have been most extensively investigated in the chemistry of the AIE behavior of boron-containing molecules. Most studies mainly focus on the development of new chelate ligands for obtaining the AIE property, and the effects of substituents on photophysical properties, such as AIE activity, luminescent color and stimuli responsivity. This section will describe typical examples of AIE- or CIE-active four-coordinate boron complexes and discuss proposed origins of the AIE nature of this class of materials.

### Boron **β**-diketonate complexes

*β*-Diketonates are one of the most widely utilized ligands for obtaining metal complexes with various metal ions. Boron *β*-diketonates are known to be versatile units for obtaining luminescent materials including polymers because of their superior luminescent properties e.g. mechanochromism, thermochromism and room-temperature phosphorescence [18–22]. However, the ACQ problem is inevitable for most of this class of complex because of their relatively planar structures resulting in undesired intermolecular interactions. Nevertheless, the AIE property has been obtained from several boron *β*-diketonates [23,24].

**Figure 3.**
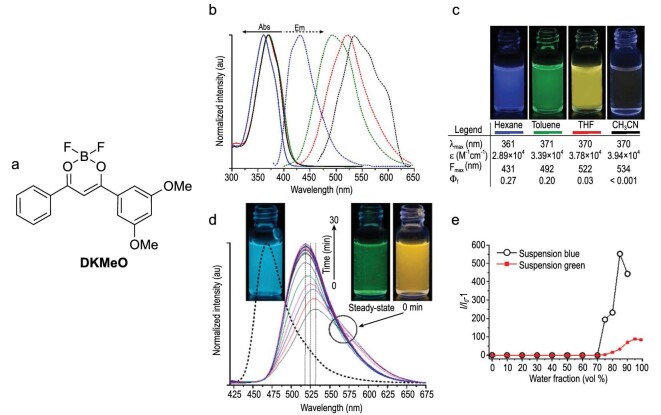
(a) The chemical structure of the AIE-active *β*-diketonate boron complex. (b) Photoelectronic spectra of DKMeO in various solvents. (c) Photographic images of DKMeO in hexane, toluene, tetrahydrofuran (THF) and acetonitrile under UV irradiation. (d) Photoluminescence spectra of blue suspension (dotted line) and time-dependent spectra of green suspension (solid lines) in 5 × 10^–5^ M CH_3_CN/water mixture at 85% and 95% fraction of water (*f*_w_), respectively. (e) A plot of relative luminescent intensity versus *f*_w_. *I*_0_ represents emission intensity in pure 5 × 10^–5^ M CH_3_CN solution. Reprinted with permission from ref [23]. Copyright 2014 American Chemical Society.

Fascinating photophysical properties were observed in dimethoxy-substituted *β*-diketonate boron complex DKMeO (Fig. 3) [23]. The luminescence from this compound showed not only an AIE property but also dependence on polymorphs, mechanochromism, thermochromism and solvatochromism. DKMeO exhibited a slight emission in the acetonitrile solution (*Φ*_f_ < 0.001, Fig. 3c), while the complex emitted much more strongly after the addition of an excess amount of water to form aggregates. Interestingly, the luminescent color was dependent on the way of adding water even if the final volume content of water was the same. When water was added dropwise to the vigorously stirred acetonitrile solutions, the obtained suspensions showed blue emission. On the other hand, the emission color was dramatically changed from yellow to green when the whole amount of water was added at once to prepare acetonitrile/water mixtures (Fig. 3d). The results of powder X-ray diffraction patterns and photophysical measurements showed that the blue and green emissions originated from the two distinct polymorphs, which are derived from the *syn*–*anti* conformers of the methoxy groups. The transient yellow-emissive suspension might be attributed to the metastable amorphous state. It should be noted that DKMeO showed relatively efficient emission in the less polar solvents (hexane, toluene and tetrahydrofuran), as shown in Fig. 3b and c. Therefore, the mechanism of the AIE phenomenon might not only be the restriction of the molecular motion but also the difference of polarity between the solution and aggregate states. By clarifying the reason why the molecular crystals of this compound are spared the problem of ACQ, far more sophisticated molecular designs are expected to be established for obtaining AIE-active materials based on *β*-diketonate complexes.

### Boron **β**-ketoiminate complexes

As mentioned above, *β*-diketonate is still not a robust structure as a scaffold of AIE-active luminophores. In contrast, it has been shown that *β*-ketoiminate ligands, also denoted as *β*-iminoenolates or *β*-enaminoketonates, are a versatile scaffold for the construction of AIE-gens [25–33]. *β*-Ketoiminate skeletons are the imine analogues of *β*-diketonate ligands, and the nitrogen atom is substituted by aryl, alkyl, silyl etc. In general, B–N bonds (∼445.6 kJ mol^–1^ or less) are weaker than B–O bonds (∼536 kJ mol^–1^) [34]. The weaker bond energy is likely to result in larger molecular motions both at the ground and excited states. Therefore, non-radiative quenching processes of the corresponding boron complexes in the solution states are probably accelerated by the replacement of oxygen to nitrogen. In solid states, such molecular motions would be restricted, and the steric hinderance of the substituent attached to the nitrogen atoms could contribute to prohibiting strong intermolecular interaction. Consequently, it could be hypothesized that boron *β*-ketoiminate complexes are likely to emit more efficiently in solid states than those in solution states.

**Figure 4.**
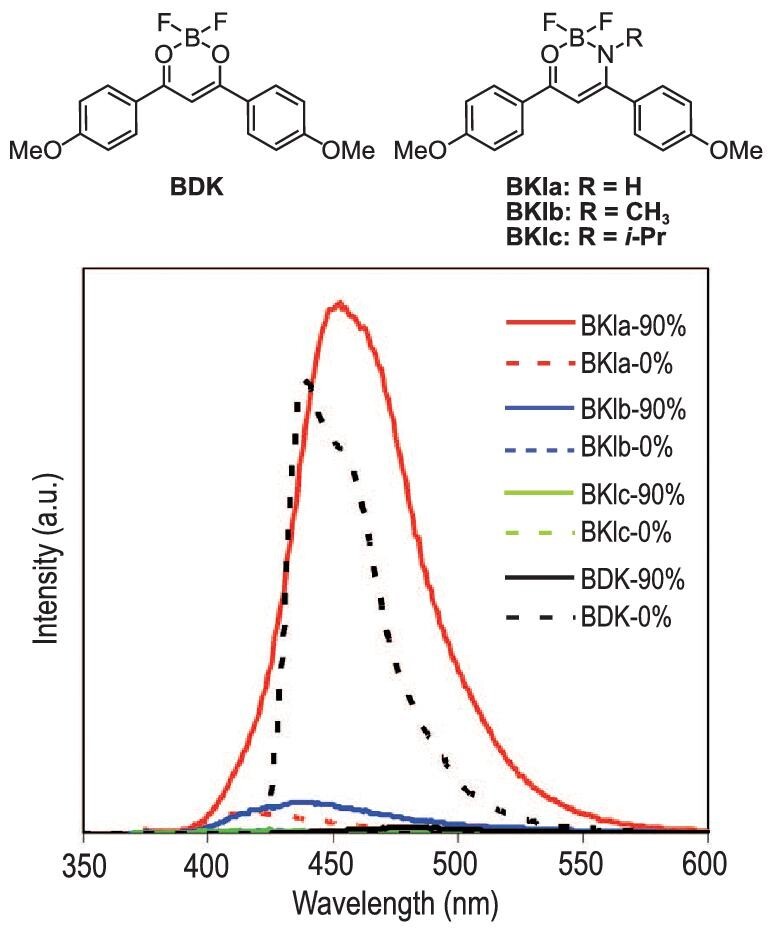
Chemical structures of *β*-diketonate and *β*-ketoiminate complexes and the dependency of photoluminescence spectra on the compositions of the solvents: THF (solid line) and THF/H_2_O (1 : 9) mixed solvent (dashed line). The concentration of each sample: 5.0 × 10^−5^  M. Reprinted with permission from ref [26]. Copyright 2013 Wiley-VCH Verlag GmbH & Co. KGaA, Weinheim. Compounds were renamed.

AIE-active boron *β*-ketoiminates have been synthesized with various types of ligands [25–33]. A typical example is shown in Fig. 4 [26]. The solid of *β*-diketonate complex BDK exhibited a decrease in photoluminescence intensity and quantum yield (*Φ*_PL_ = 0.36) compared to those of the solution (*Φ*_PL_ = 0.91). On the other hand, the corresponding boron *β*-ketoiminates, BKIa, BKIb and BKIc, showed drastic emission enhancement caused by aggregation (${\mathit \Phi}_{\rm PL}^{\rm {solid}}$: 0.76 for BKIa; 0.42 for BKIb; 0.30 for BKIc), while the emissions from their solution samples were very weak (${\mathit \Phi}_{\rm PL}^{\rm {solution}}$ ∼ 0.01). Under frozen and viscose conditions, the emission intensity of these complexes increased compared to that in the solutions. These results support that intramolecular motions should accelerate non-radiative quenching paths in solution at room temperature.

**Figure 5.**
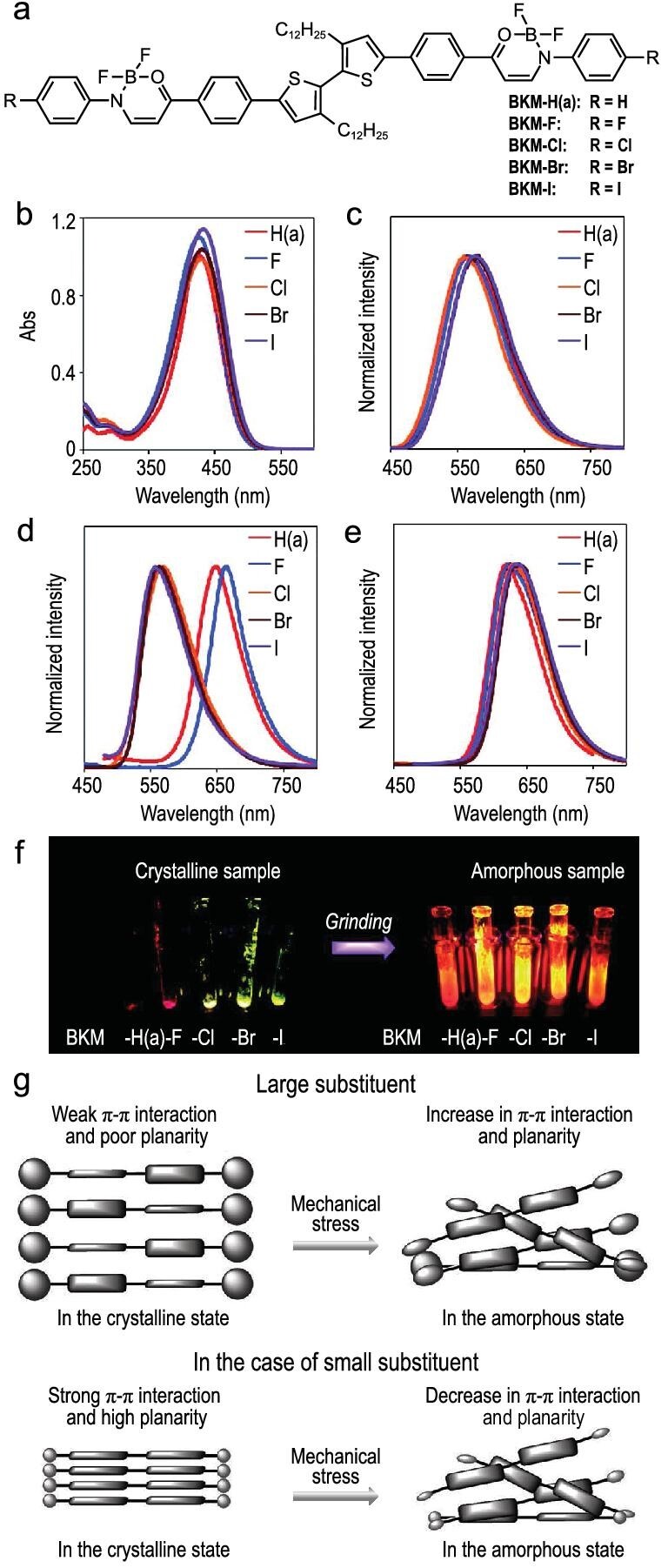
(a) Chemical structures of the triads composed of the boron *β*-ketoiminate core and the various substituents. (b) UV–Vis absorption, and (c) normalized photoluminescence spectra of the complexes in THF (1 × 10^–5^ M). Normalized photoluminescence spectra of the complexes in (d) the crystalline states, and (e) after grinding. (f) Photographs of the complexes in the crystalline (left) and amorphous (right) states under UV (365 nm) irradiation. (g) Illustration of a plausible mechanism of the effect of the substituents on the mechanochromic fluorescence. Reprinted with permission from ref [44]. Copyright 2015 Wiley-VCH Verlag GmbH & Co. KGaA, Weinheim. Compounds were renamed.

Since AIE-active boron *β*-ketoiminates have relatively high planarity, it is expected that the extension of the π-electronic planes could effectively modulate their photophysical properties. Mechanical stimuli, such as pressing, crushing and grinding, occasionally change the luminescent color of crystalline samples of solid-state emissive molecules. This property is called mechano- or piezochromic luminescence and is expected to be utilized for pressure sensors and optical recording/memory devices [35–43]. The triads, BKM, containing two boron *β*-ketoiminates and the bithiophene linker with or without substituents at both ends, were synthesized (Fig. 5a) [44]. All the compounds showed similar electronic absorption and photoluminescence spectra in the solution states (Fig. 5b and c). Meanwhile, in the crystalline powder, the triads containing the smaller substituents, BKM-H(a) and BKM-F, provided yellow fluorescence. The compounds have relatively larger substituents, BKM-Cl, BKM-Br and BKM-I, exhibiting red fluorescence (Fig. 5d). Interestingly, mechanical grinding leads to hypsochromic and bathochromic shifts of the emission bands for the former group (BKM-H(a) and BKM-F) and the latter group (BKM-Cl, BKM-Br and BKM-I), respectively (Fig. 5e). The initial emission colors were recovered by heating the ground samples. From the results of powder X-ray diffraction and differential scanning calorimetry before and after the mechanical treatment, the grinding process increased the content of the amorphous domain of the samples. These observations suggested the following plausible mechanism of the contrary mechanochromic behavior depending on the kind of substituents (Fig. 5g): in the case of triads having smaller substituents, the molecules could interact with a face-to-face motif and realize tight packing structures in the crystalline states. As a result, emission bands appeared in the relatively longer-wavelength regions in the initial crystalline states. By collapsing such tight packing, random molecular distributions should be obtained, resulting in the hypsochromic shifts. On the other hand, the steric hindrance of the larger substituents could disturb such a face-to-face stacking structure in the crystals. In the amorphous domains, π–π interactions could be more frequently facilitated, leading to the bathochromic shifts. This is one example that offers environment-sensitive solid-state emission of AIE-active complexes.

### Boron **β**-diketiminate complexes

*β*-Diketiminate ligands, also known as *β*-diiminates, are the aza analogs of *β*-diketonate ligands as well as *β*-ketoiminate ligands. ‘Ketimine’ is the name of an imine analogous to a ketone, not to an aldehyde. ‘*β*-Ketoimine’ is the name of an imine possessing a ‘keto’ group at the *β* position of its imino group. Boron complexes of *β*-diketiminates are also likely to be AIE-active materials like *β*-ketoimine complexes, because both the oxygen atoms in *β*-diketonates are replaced by a nitrogen atom with a bulky and movable aryl group. Additionally, different types of substituents can be introduced independently into each aryl group of *β*-diketiminate ligands. Consequently, these ligands have been utilized in order to isolate the classes of unstable main-group and transition metal complexes composed of the group 13 elements, which had not been accessible using other bidentate ligands [45–54]. On the other hand, there have been a limited number of studies about the photophysical and electrochemical properties of these complexes, probably because the π-conjugation lengths of widely used *β*-diketiminate ligands are localized at the NCCCN and the corresponding complexes are usually colorless. However, it could be envisioned that *β*-diketiminate ligands would enable us to access functional optoelectronic materials and create possible applications of many kinds of metal complexes that have not been obtained by other ligands.

**Figure 6.**
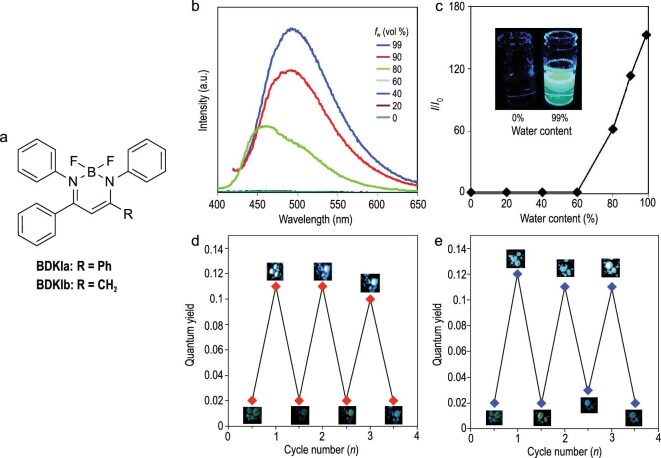
(a) Chemical structures of boron *β*-diketiminate complexes. (b) Photoluminescence spectra of BDKIa in the acetonitrile/H_2_O mixture (5 × 10^−5^  M) with various water contents (*f*_w_). (c) Dependence of intensity ratio of BDKIa on *f*_w_. Repeatability of the emission behavior of BDKIb between amorphous and crystalline states by (d) fuming–melt/quenching and (e) heating–melt/quenching cycles (see text for the detail). Reprinted with permission from ref [56]. Copyright 2014 Wiley-VCH Verlag GmbH & Co. KGaA, Weinheim. Compounds were renamed.

In an early study in 2008, it was reported that a boron *β*-diketiminate complex with an extended π-conjugation is fluorescent in the visible region. Importantly, it was clarified that the emission intensity of the complex is enhanced by aggregation, but the photoluminescence quantum yields in the solid state were not evaluated [55]. In later reports, it was reported that the luminescence quantum yields of the boron *β*-diketiminate complexes, BDKIa and BDKIb (Fig. 6a), were higher in the crystalline states (*Φ*_PL_ = 0.23 for BDKIa and 0.11 for BDKIb) than those in the amorphous (*Φ*_PL_ = 0.02 for both compounds) and solution states (*Φ*_PL_ < 0.01 for both compounds) [56]. The crystalline aggregates can be obtained by reprecipitation from the mixture of acetonitrile solutions and water (Fig. 6b and c). The amorphous solids were prepared by rapid quenching of the melt samples (a melt/quenching process). The amorphous samples were converted to the crystalline sample by fuming the dichloromethane vapor for 30 minutes (a fuming process), or heating above the crystallization temperature (a heating process). Consequently, the crystalline and amorphous states of these complexes were interconverted repeatedly by means of the fuming–melt/quenching or heating–melt/quenching cycles (Fig. 6d and e). It was proposed that the packing structures in their crystalline states should play a critical role in the restriction of molecular vibrations and motions leading to non-radiative quenching paths. Since amorphous states are generally sparser than crystalline states, radiationless processes may be accessible enough to completely quench the excited energy without strong emission in the amorphous states. Therefore, the CIE property was observed in the boron *β*-diketiminates. Another group reported the AIE behavior of the similar boron complexes with fused ring structures at around the same time [57]. After these observations, the chemistry of the emission from boron *β*-diketiminates emerged [58–63].

**Figure 7.**
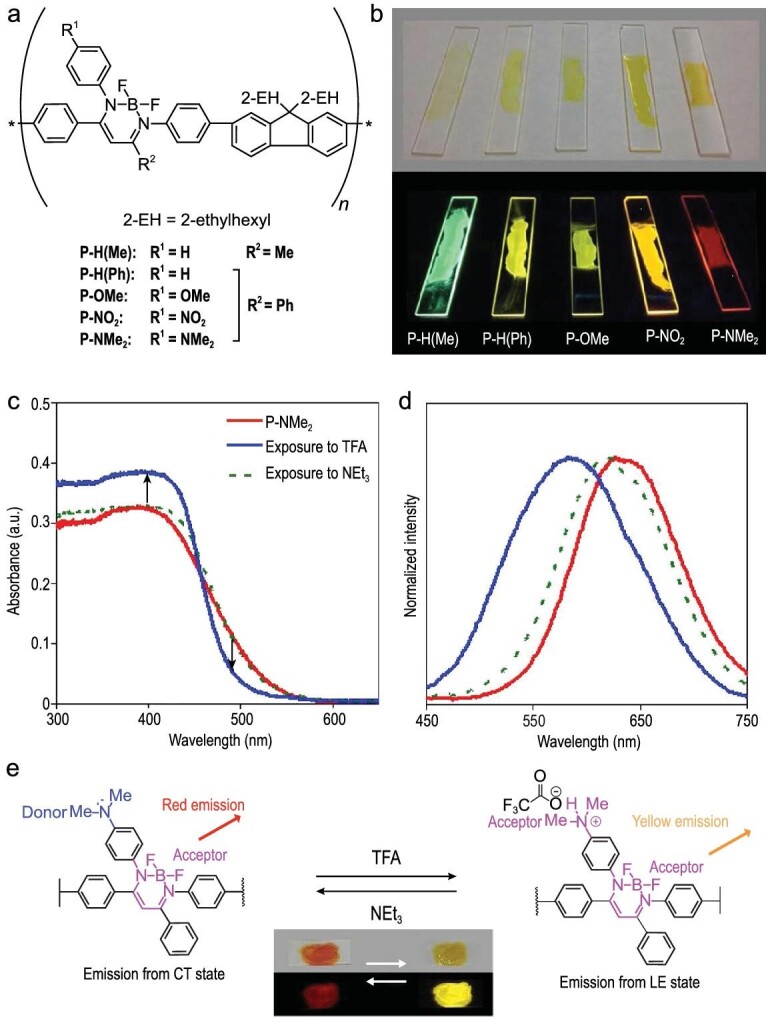
(a) Chemical structures of the conjugated polymers containing *β*-diketiminate complexes. (b) Photographs of the drop-cast films of the polymers formed on quartz substrates. (c) UV–Vis absorption and (d) photoluminescence spectra of P-NMe_2_ before and after exposure to TFA and NEt_3_ vapor in the thin-film state upon excitation at 391 nm. (e) Plausible mechanism of acid-base sensing. Reprinted with permission from ref [58]. Copyright 2014 American Chemical Society.

Suzuki-Miyaura cross-coupling polycondensation between dihalogenated boron *β*-diketiminates and the corresponding comonomers successfully afforded AIE-active π-conjugated polymers [58]. The emission colors of the drop-cast films of the polymers are dependent on the electronic property of the substituent of the ligand (Fig. 7a and b). Complexes with an electron-donating substituent showed a bathochromic shift of their emission band compared to the non-substituted compounds. The electron-withdrawing substituents, on the other hand, hypsochromically shifted the luminescence spectra. Based on these observations, film-type luminescent sensors for acid-base [58] or redox [64] reactions were developed using this class of conjugated polymers. In the acid-base sensing system (Fig. 7c and d) [58], the emission properties were altered by acidification of the polymer films composed of the dimethylamino groups (P-NMe_2_). Before treatment with the vapor of trifluoroacetic acid (TFA), the spin-coat films on quarts substrates of the polymer exhibited red emission. After the treatment of the films with the vapor of TFA, the emission color turned to yellow because the protonated ammonium groups acted as electron-withdrawing groups. Treatment with the triethylamine vapor recovered the initial red emission.

### BODIPY-based systems

**Figure 8.**
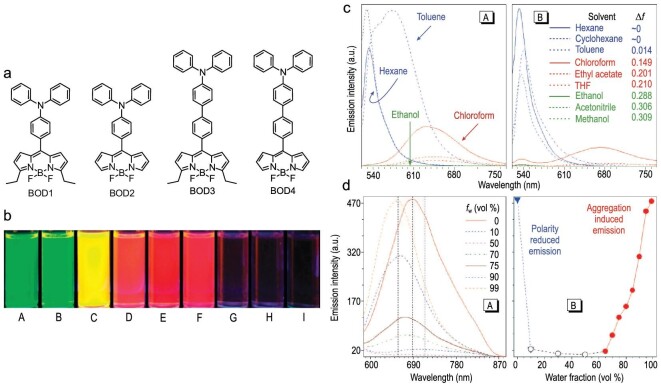
(a) Chemical structures of the BODIPY-based donor–acceptor luminogens. (b) Photographs of BOD1 under UV irradiation in various solvents (A: hexane; B: cyclohexane; C: toluene; D: chloroform; E: ethyl acetate; F: THF; G: ethanol; H: acetonitrile; I: methanol). (c) Photoluminescence spectra of (A) BOD1 and (B) BOD2 in the various solvents. (d) (A) Photoluminescence spectra and (B) emission intensities of BOD3 in THF/water mixtures with different water contents. Reprinted with permission from ref [65]. Copyright 2009 American Chemical Society.

Much effort has also been made to construct AIE-active systems based on BODIPY dyes, which are usually considered to be ineffective emitters in the solid states due to their planar structure as mentioned in the Introduction [65–69]. One of the incipient examples was reported in 2009 for donor–acceptor type luminogens (Fig. 8a) [65]. These molecules are composed of a diphenylamino group as an electron donor and a BODIPY moiety as an electron acceptor with one or two phenylene spacers. These compounds show a twisted intramolecular charge transfer (TICT) property. They exhibit green emission derived from the locally-excited states in the low-polarity solvents, while the emission color changes to red, originating from the TICT state when the solvent polarity increases (Fig. 8b and c). The intensities of the red TICT emissions in tetrahydrofuran (THF)/water mixtures are strongly dependent on the contents of water (*f*_w_). When *f*_w_ is low (<60 vol%), the red emissions become negligible due to the very high hydrophilicity of water. In contrast, these molecules show their TICT emission when a large amount of water is added (*f*_w_ > 70%) because the solutions cannot solvate the dyes and nanoaggregates are formed. In the case of BOD3 and BOD4, it is of interest to note that the emissions are enhanced compared to those in the pure THF solutions (Fig. 8d). Their intramolecular motions are likely to be restricted by the solidification.

**Figure 9.**
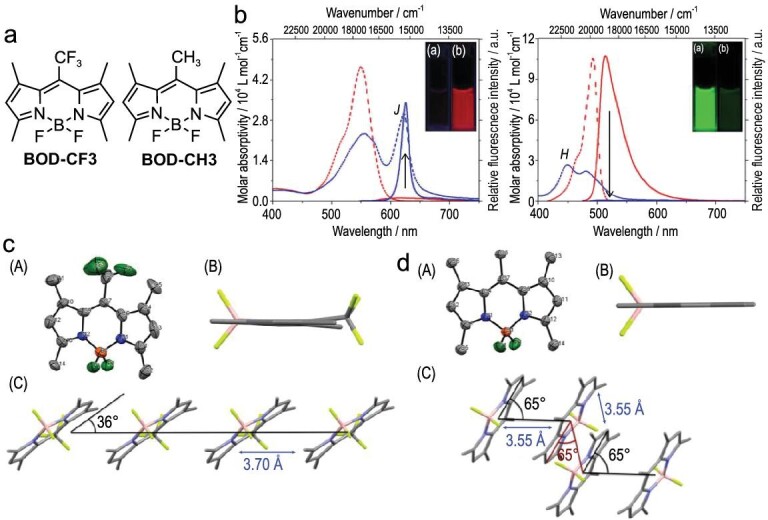
(a) Chemical structures of the *meso*-substituted BODIPYs. (b) Absorption and emission spectra of BOD-CF3 (left) and BOD-CH3 (right). Single-crystal structures of (c) BOD-CF3 and (d) BOD-CH3. Reproduced from ref [66] with permission from The Royal Society of Chemistry.

Another exciting example was constructed with *meso*-trifluoromethyl-substituted BODIPY BOD-CF3 (Fig. 9a) [66]. The emission intensity of BOD-CF3 in the solution states is significantly lower (*Φ*_PL_ = 0.003 in acetonitrile) than that of the methyl counterpart BOD-CH3 (Fig. 9b). Such weak luminescence may originate from the large structural difference between the S_0_ and S_1_ states resulting in the small Franck-Condon factor for the electronic transition. Surprisingly, the emission intensity of BOD-CF3 is enhanced as the volume fraction of water (*f*_w_) of the acetonitrile/water mixtures increases. The colloidal suspension of the BOD-CF3 with 99% of *f*_w_ shows sharpened absorption and emission spectra with a significantly small Stokes shift (51 cm^–1^; 2 nm) compared to the acetonitrile solution (Fig. 9b). In sharp contrast, the aggregation causes the annihilation of the emission of BOD-CH3. The results of the single-crystal X-ray analysis revealed that the transition dipole moments of BOD-CF3 are aligned with 36º, while those of BOD-CH3 are aligned with 65º (Fig. 9c and d). These results clearly indicate that the formation of J-aggregation makes an efficiently emissive path in the BOD-CF3 aggregates, leading to a strong AIE effect. BOD-CH3, on the other hand, exhibits the H-type aggregation resulting in the optically dark states. Solid-state luminescent materials based on BODIPY dyes with such a J-aggregation character have attracted broad attention [68–74].

### Other four-coordinate boron complexes and origins of AIE properties

**Figure 10.**
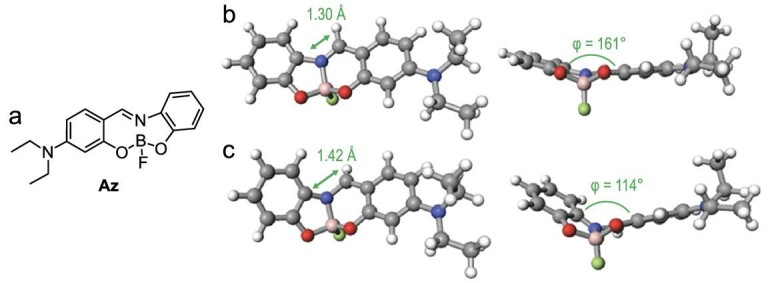
(a) The chemical structure of the azomethine complex Az. The optimized geometries of Az in (b) the S_0_ and (c) the S_1_ states. Optimization calculations were carried out at the B3LYP/6–311G^**^ level for the ground state and the time-dependent B3LYP/6–311+G^**^ level for the excited state. Reprinted with permission from ref [75]. Copyright 2017 Wiley-VCH Verlag GmbH & Co. KGaA, Weinheim.

Most AIE-active molecules possess peripheral phenyl groups and rotative bonds, which probably accelerate non-radiative processes in the solution states, leading to emission annihilation. On the other hand, several boron complexes without such structural features have shown AIE behavior [75]. As an interesting example, the photophysical properties of an azomethine complex, Az, are described here (Fig. 10a). Its optimized structures were estimated using a series of density functional theory (DFT) calculations in advance. It was implied that the complex would possess planar structure because of its fused structure (Fig. 10b). Indeed, the structure determined from the analysis of the single-crystal X-ray diffraction of the synthesized complex is almost identical to the calculated structure. Interestingly, the optimized structure in the first singlet excited state (S_1_) suggested drastic structural bending during structural relaxation (Fig. 10c). This structural bending should give rise to the localization of π-electrons and radiationless decay of the excited energy. Hence, it could be hypothesized that Az is likely to show the AIE property because aggregation would restrict the large relaxation process.

To evaluate the justification of our presumption, we synthesized Az [75]. Optical measurements revealed both the AIE and CIE properties of Az. A solution of Az shows a large Stokes shift at room temperature. This result indicates that a large structural relaxation should occur in the S_1_ state. The fluorine atom protruding from the molecular plane seems to repel strong π–π interactions. These data strongly encourage us to design a solid-state luminescence system based on boron complexes with such excited-state flexibility.

**Figure 11.**
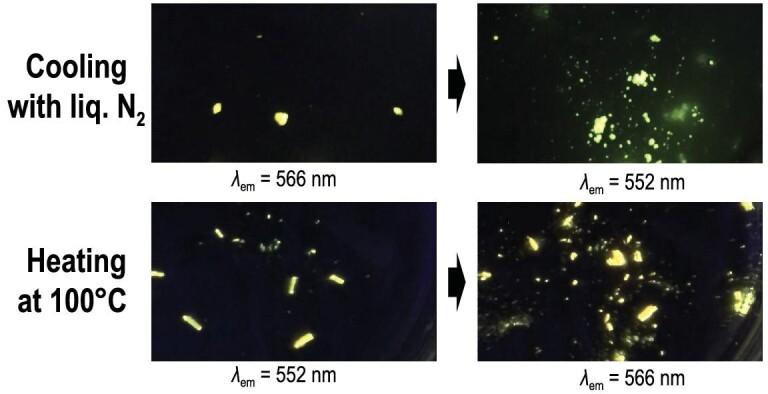
Photographs of the crystals of Az before/after cooling with (top) liquid nitrogen or (bottom) heating to 100ºC under UV irradiation. Fracture of the crystals and emission color change can be observed. Reprinted with permission from ref [75]. Copyright 2017 Wiley-VCH Verlag GmbH & Co. KGaA.

Additionally, it is of interest to note that temperature alteration causes unique mechanical movements and chromic behavior of the crystals of Az (Fig. 11). This complex was crystallized in the two distinct polymorphs, which exhibited green and yellow emission. Differential scanning calorimetry (DSC) clearly indicated that these two polymorphs thermally transformed each other. Surprisingly, mechanical movements such as hopping or fragmentation of the crystals were observed during heating and cooling. Such motion induced by thermal stimuli is known as the thermosalient effect. The results of powder X-ray diffraction analysis revealed that the crystal structure changes at the same time the thermosalient effect is observed. Moreover, this transition is also accompanied by the chromism of the appearance and the emission. The ‘flexibility’ of the boron complex might make its crystal sparser and tolerate the mechanical motions during the crystal transition.

Bending motions of four-coordinate boron complexes upon electronic excitation have been estimated in AIE-active boron difluorohydrazones [76]. A series of quantum calculations suggested that the bending motion of these molecules, named ‘flip-flop’, could lead the excited molecules to a conical intersection through which excitons non-radiatively quench. On the other hand, this class of complexes also possess rotative aryl groups. Other research has suggested that the restriction of the rotational motions of the peripheral aromatic ring could suppress the internal conversion of the excitons to the ‘dark’ S_1_ state [77]. As shown above, the origins of the AIE and CIE properties are still not clear. Much more experimental and theoretical studies are required for a comprehensive understanding of these properties.

## OTHER GROUP 13 ELEMENTS

Studies of luminescent properties of four-coordinated compounds containing heavier group 13 elements are still rare. This is probably because the heavy group 13 elements such as Al and Ga prefer the five- or six-coordinated structures and the four-coordinated structures are usually unstable. Nevertheless, the construction of optical materials based on the heavy elements has been an attractive research topic since four-coordinated heavy elements promise to provide unique bonding and electronic nature [14,78–80]. In this section, we will provide a brief overview of aluminum- and gallium-containing AIE luminogens composed of *β*-diketiminate ligands. It is of interest to note that a few examples of AIE-active complexes [81] or metal–organic frameworks [82] have emerged. There is, of course, much room for development of photo-functional materials based on indium [83–85].

### Aluminum

**Figure 12.**
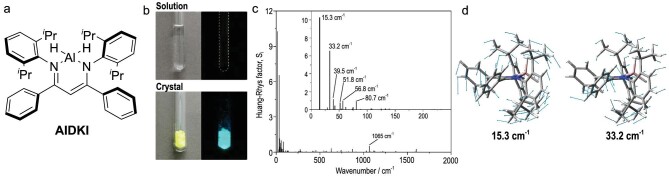
(a) The chemical structure of an aluminum *β*-diketiminate complex. (b) Photographs of the solution and crystalline states of AlDKI under natural light and UV (365 nm) irradiation. (c) Huang–Rhys factor, *S_i_*, for each normal mode of AlDKI calculated for the S_0_–S_1_ transition. (Inset: low-frequency region.) Normal modes with relatively large HR factors (*S_i_* > 0.5) are labeled with their wavenumber. (d) Displacement vectors attributed to the normal modes (left) *ω*_1_ (15.3 cm^−1^) and (right) *ω*_3_ (33.2 cm^−1^). Reprinted with permission from ref [86]. Copyright 2019 MDPI (Basel, Switzerland).

The AIE and CIE nature of an aluminum-containing compound was reported for the dihydridoaluminum complex of a *β*-diketiminate ligand (Fig. 12a) [86]. This complex showed typical CIE behavior as well as a series of boron *β*-diketiminate complexes (Fig. 12b). From the results of DFT calculations, large structural relaxation, such as bending, was hardly observed. In addition, the phosphorescence quantum yield (∼0.10) was considerably lower than the fluorescence quantum yield (∼0.90) even at 77 K. Thus, we postulated that the non-radiative decay process in the solution state would be mainly attributed to internal conversion from S_1_ to S_0_. To estimate the contributions of each normal vibrational mode to the internal conversion, we calculated Huang–Rhys (HR) factors for the transition between S_0_ and S_1_ (Fig. 12c). The larger value of HR factors should indicate the larger contribution of the corresponding vibration to the radiationless quenching. The results of a series of DFT calculations suggested that the two types of vibrational modes, out-of-plane bending (Fig. 12d, left) and rotation of the peripheral aromatic rings (Fig. 12d, right), might play a pivotal role in the internal conversion process of the excited molecules. Importantly, this is the first report to assign the out-of-plane bending mode to a key factor of the AIE and CIE behavior of the complexes composed of the group 13 elements.

**Figure 13.**
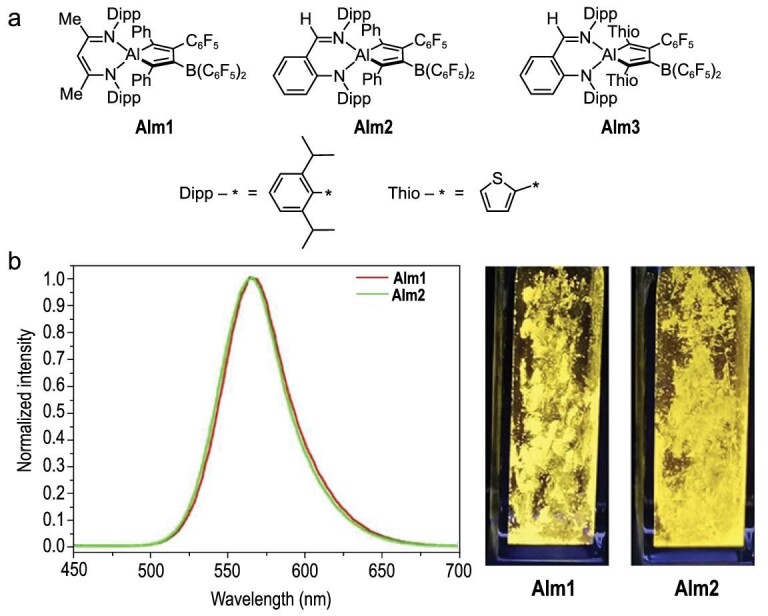
(a) Chemical structures of alumoles Alm1 and Alm2. (b) Normalized photoluminescence spectra and photographs of the solids of Alm1 and Alm2 under UV-light irradiation 365 nm. Reprinted with permission from ref [87]. Copyright 2020 the authors. Published by Wiley-VCH Verlag GmbH & Co. KGaA. Compounds were renamed.

AIE-active aluminacyclopentadienes, named alumoles, have been reported recently. Alumoles can be regarded as the aluminum analog of siloles, which are important AIE-gens. Three ligand-stabilized alumoles, Alm1, Alm2 and Alm3 (Fig. 13a), were synthesized in the reaction between the dialkynyl complex LAl(CCR)_2_ and tris(pentafluorophenyl)borane [87]. Interestingly, alumoles Alm1 and Alm2 showed typical AIE behavior. Their fluorescence emission bands in benzene were very weak, whereas they exhibited bright yellow emissions in the solid state (Fig. 13b). The luminescence quantum yields of Alm1 and Alm2 were estimated to be 0.093% and 0.086%, respectively. In Alm1 and Alm2, there are six rings attached to the alumole core with two Ph, three C_6_F_5_ and one AlN_2_C_3_. These peripheral rings might be essential for AIE behavior. On the other hand, the alumole Alm3, which is the thienyl analog of Alm2, is hardly emissive in both solid and solution. The less planar central alumole ring might result in such annihilation of emission. The *N*,*N*-chelate ligand structures might also play a crucial role since such structures can be frequently found in the various AIE-active complexes. These promising results encourage us to develop novel AIE-gens constituted by the other group 13 heteroles, such as boroles and galloles.

### Gallium

**Figure 14.**
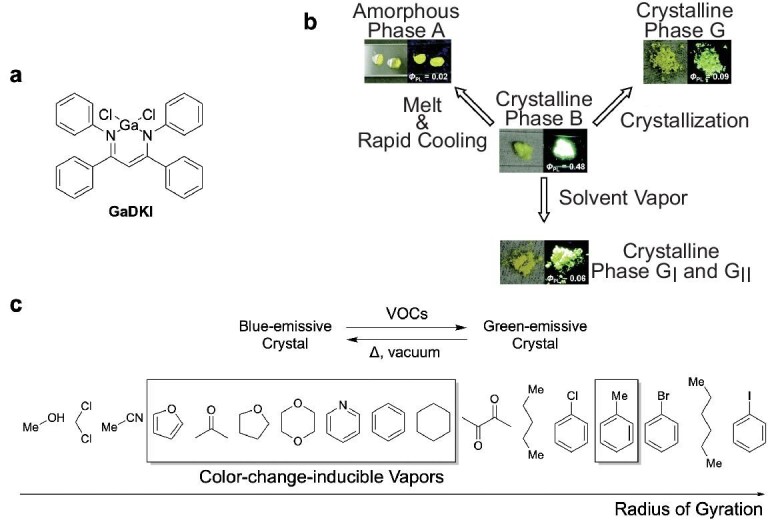
(a) The chemical structure of a gallium *β*-diketiminate complex. (b) Photographs of the complexes in each state and the stimuli-responsive behavior. Reproduced from ref [59] with permission from The Royal Society of Chemistry. (c) Schematic representation of the vapochromic luminescence. Compound was renamed.

Gallium has been employed as a central element of typical (semi-)metal complexes instead of boron. It can be envisioned that the larger ionic radius of gallium may induce sparse packing of gallium-based complexes in the crystalline states. Therefore, photophysical properties of gallium complexes could differ completely from the corresponding boron complexes. Indeed, the crystals of a gallium *β*-diketiminate complex (GaDKI, Fig. 14a) exhibited more efficient photoluminescence than the corresponding boron complex [59]. This enhancement of the luminescence might result from the relatively weak intermolecular electronic interactions in the crystalline state of the gallium complex. It is of interest to note that solvent-vapor annealing brings about the emission-color change accompanied by the crystal–crystal phase transition (Fig. 14b and c). When the crystalline powder is exposed to the vapor of specific volatile organic compounds (VOCs), the color of fluorescence changes rapidly from blue to green. Surprisingly, only the VOCs with a limited range of radius of gyration (1.75–2.02 Å) induce such chromic fluorescence with crystal–crystal transition. The other evaluated parameters of the VOCs, vapor pressure and dipole moment, are independent of whether the chromism occurs or not. Such a transition might be realized by the introduction of flexibility in the crystal originating from the weak gallium–nitrogen coordination. With regard to these results, *β*-diketiminate complexes have attracted attention for developing functional solid-state luminescent materials. Additionally, it is worth noting that GaDKI is the first example of the AIE- and CIE-active gallium complex.

**Figure 15.**
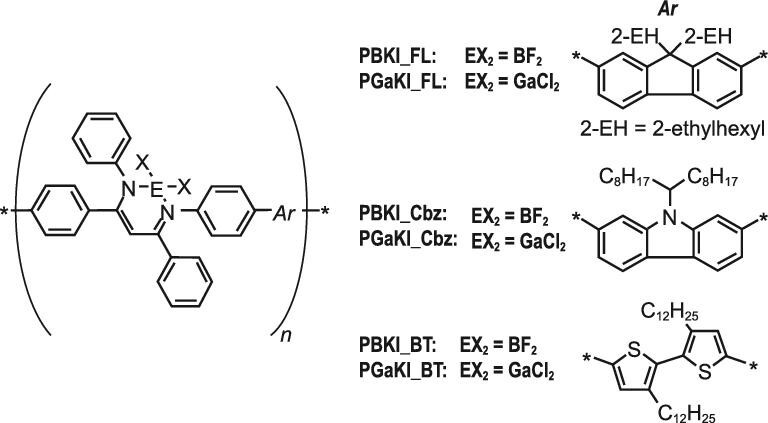
Chemical structures of π-conjugated polymers composed of boron or gallium *β*-diketiminate complexes and three distinct comonomers: FL: 2,7-fluorenyl; Cbz: 2,7-carbazolyl; BT: 5,5’-bithiophenyl.

π-Conjugated polymers containing gallium *β*-diketiminates, PGaKI_FL, PGaKI_Cbz and PGaKI_BT (Fig. 15), were synthesized through the post-complexation methodology with the corresponding polymeric ligands [61]. Photophysical, electrochemical and theoretical studies suggested that the lowest unoccupied molecular orbitals (LUMOs) of the gallium-containing polymers should be lying at lower energy levels than those of the corresponding boron polymers, PBKI_FL, PBKI_Cbz and PBKI_BT (Table 1). Meanwhile, their highest occupied molecular orbitals (HOMOs) stayed at a similar level. As a result, the gallium polymers exhibited the AIE behavior in a lower energy region than the boron polymers. It was also implied that the synthetic approach through polymeric ligands should provide a versatile way to access functional polymers composed of complexes with other heavy elements.

**Table 1.** Photophysical and electronic properties of the polymers.

	${\mathit \lambda}_{\rm abs}^{\rm {solution}}$ /nm	${\mathit \lambda}_{\rm PL}^{\rm {film}}$ nm	${\mathit \Phi}_{\rm PL}^{\rm {solution}}$ ${\mathit \Phi}_{\rm PL}^{\rm {film}}$	*E*_HOMO_/eV^a^	*E*_LUMO_/eV^a^
PBKI_FL	399	545	<0.01	–6.27	–3.53
			0.07		
PBKI_Cbz	397	552	<0.01	–6.18	–3.44
			0.07		
PBKI_BT	404	575	<0.01	–6.10	–3.45
			0.07		
PGaKI_FL	411	575	<0.01	–6.19	–3.60
			0.05		
PGaKI_Cbz	410	573	<0.01	–6.15	–3.54
			0.05		
PGaKI_BT	420	601	<0.01	–6.08	–3.54
			0.03		

^a^Calculated from the results of cyclic voltammetry and optical band gap (${\mathit E}_{\rm g}^{\rm {opt}}$) with the following empirical formula: *E*_HOMO_/eV = *E*_LUMO_/eV – ${\mathit E}_{\rm g}^{\rm {opt}}$/eV, *E*_LUMO_/eV = – *E*_red_/eV – 4.80.

## CONCLUSION

Recent advances in the development of AIE-active complexes composed of the group 13 elements are reviewed. Various types of stimuli-responsive chromic materials, as well as solid-state emissive molecules, have become accessible by AIE-active element blocks. Additionally, it is anticipated that ‘flexibility’ of boron complexes at electronically excited states is likely to be one of the key factors in the AIE property of the complexes. In order to detect tiny stimuli and slight environmental changes, we still require enhancement of sensitivity in optical sensors. Therefore, AIE-active element blocks based on the group 13 elements with novel chemical structures would be a scaffold for designing and constructing advanced sensing materials to meet these demands. Furthermore, the number of examples of AIE- and CIE-active compounds composed of aluminum or gallium has been gradually increasing. In addition, a few examples of AIE-active systems based on indium complexes have emerged. Further development of these sprouting studies must enable the heavier elements to show their stuff as next-generation functional optoelectronic materials.
